# Autophagy dysfunction and regulatory cystatin C in macrophage death of atherosclerosis

**DOI:** 10.1111/jcmm.12859

**Published:** 2016-04-14

**Authors:** Wei Li, Nargis Sultana, Nabeel Siraj, Liam J Ward, Monika Pawlik, Efrat Levy, Stefan Jovinge, Eva Bengtsson, Xi‐Ming Yuan

**Affiliations:** ^1^ Division of Obstetrics and GynaecologyDepartment of Clinical and Experimental MedicineFaculty of Health SciencesLinköping UniversityLinköpingSweden; ^2^ Occupational and Environmental Medicine CenterHeart and Medicine CenterCounty Council of ÖstergötlandLinköpingSweden; ^3^ Department of internal medicineUniversity of Alberta EdmontonAlbertaCanada; ^4^ Nathan S. Kline Institute for Psychiatric ResearchOrangeburgNYUSA; ^5^ Nathan S. Kline Institute for Psychiatric ResearchDepartments of Psychiatry and Biochemistry and Molecular PharmacologyNew York University Langone School of MedicineNew YorkNYUSA; ^6^ Department of Clinical SciencesSkåne University HospitalLund UniversityLundSweden

**Keywords:** autophagy, cystatin C, macrophage cell death, lysosomal membrane permeabilization

## Abstract

Autophagy dysfunction in mouse atherosclerosis models has been associated with increased lipid accumulation, apoptosis and inflammation. Expression of cystatin C (CysC) is decreased in human atheroma, and CysC deficiency enhances atherosclerosis in mice. Here, we first investigated the association of autophagy and CysC expression levels with atheroma plaque severity in human atherosclerotic lesions. We found that autophagy proteins Atg5 and LC3β in advanced human carotid atherosclerotic lesions are decreased, while markers of dysfunctional autophagy p62/SQSTM1 and ubiquitin are increased together with elevated levels of lipid accumulation and apoptosis. The expressions of LC3β and Atg5 were positively associated with CysC expression. Second, we investigated whether CysC expression is involved in autophagy in atherosclerotic apoE‐deficient mice, demonstrating that CysC deficiency (CysC^−/−^) in these mice results in reduction of Atg5 and LC3β levels and induction of apoptosis. Third, macrophages isolated from CysC^−/−^ mice displayed increased levels of p62/SQSTM1 and higher sensitivity to 7‐oxysterol‐mediated lysosomal membrane destabilization and apoptosis. Finally, CysC treatment minimized oxysterol‐mediated cellular lipid accumulation. We conclude that autophagy dysfunction is a characteristic of advanced human atherosclerotic lesions and is associated with reduced levels of CysC. The deficiency of CysC causes autophagy dysfunction and apoptosis in macrophages and apoE‐deficient mice. The results indicate that CysC plays an important regulatory role in combating cell death *via* the autophagic pathway in atherosclerosis.

## Introduction

Accumulation of oxidized lipids and apoptotic cells in atherosclerotic lesions contributes to plaque rupture and clinical complications, including myocardial infarction and stroke 1. In atherosclerotic disease, lipids are accumulated and oxidized in the intima of the vessel wall. Oxidized lipids are taken up by macrophages that become foam cells. Oxidized lipids in lysosomes of macrophages not only cause expansion of lysosomal compartments 2 but also increase lysosomal pH 3 and alter lysosomal functions and enzyme activity 4, 5, 6, 7. All these result in further accumulation of toxic materials within atheroma lesion including oxidized lipids and un‐degradable material, followed by cellular stress and macrophage cell death 8. Consequently, macrophage apoptosis results in intimal necrosis and promotes destabilization of advanced lesions. In this process, the accumulation of oxidized lipids may also lead to induction of autophagy 9, 10, 11, 12.

Autophagy is a lysosomal degradation pathway for cytoplasmic material and is activated under stress conditions 8. Autophagy can be a cell survival mechanism by generating free amino acids and fatty acids to maintain cellular function. However, it can also promote cell death through excessive self‐digestion and degradation of essential cellular constituents 13. In the autophagy process, a pathway of apoptotic cell death has been described, which involves lysosomal disruption with release of proteolytic enzymes into the cytosol 14. Cystatin C (CysC) is an important endogenous inhibitor of cathepsins B, H, K, L and S. Recent work suggests that CysC may play a protective role in neurodegenerative diseases and cerebral vasospasm by inducing autophagy 15. Increasing endogenous and exogenous CysC decreased brain ischaemia in rats *via* activating autophagy pathways 16, 17. Expression of CysC is decreased in human atheroma 18, and CysC deficiency enhances atherosclerosis 19. At present, the role of CysC and its relation to autophagy and apoptotic cell death in atherosclerosis has not been investigated.

In this study, we first examined the expression of the autophagy markers LC3β, Atg5, p62/SQSTM1 and ubiquitin in human carotid plaques and their relation to CysC expression, lipid accumulation and atheroma plaque severity. Second, we investigated the role of CysC in autophagy and apoptosis in ApoE‐deficient mice and in macrophages isolated from CysC^−/−^ or WT mice. Third, we studied effect of exogenous CysC on lipid accumulation in 7‐oxysterol‐treated macrophages.

## Materials and methods

### Cell culture

Human monocytic THP‐1 cells were maintained in RPMI‐1640 culture medium with 10% foetal bovine serum in a humidified atmosphere (5% CO_2_) at 37°C and subcultivated twice a week. In some experiments, THP‐1 cells were differentiated into macrophages after incubation with phorbol myristate acetate (300 nM) for 24 hrs. After being washed with culture medium, the cells were further cultured for 24 hrs under standard culture conditions before experiment initiation.

For experiments, the above cells were treated with a mixture of 7β‐hydroxycholesterol (7βOH) and 7keto‐cholesterol (7keto; Sigma‐Aldrich, St. Louis, MO, USA) at a ratio of 1:1.8 (2mix) for 12, 24 or 48 hrs (28 μM) as described previously 20. Cells treated with cholesterol or ethanol were used as controls. In some experiments, cells were exposed to recombinant CysC (Sigma‐Aldrich) at 1–2 μg/ml together with 2mix for 24 hrs. After treatment, cells were collected and analysed as described below.

### Peritoneal macrophages from CysC knockout or wild‐type mice

Homozygous CysC‐deficient (CysC^−/−^) mice described by Huh *et al*. 21 were kindly provided by Dr. Anders Grubb, Lund University Hospital, Lund, Sweden 19. All animal procedures were performed following the National Institutes of Health guidelines with approval from the Institutional Animal Care and Use Committee at the Nathan S. Kline Institute for Psychiatric Research. All efforts have been made to minimize animal suffering and the numbers of mice used.

Resident peritoneal cells were prepared from 7‐ to 9‐week‐old CysC^−/−^ and wild‐type mice (CysC^+/+^) of both sexes according to the basic protocol as described by Zhang *et al*. 22. Briefly, the mice were killed by cervical dislocation exposing them to a minimum of stress. Under sterile conditions, their peritoneal cavities were filled with cold Dulbecco's without magnesium and calcium (Gibco Life Technologies, New York City, NY, USA) and the fluid reaspirated with a 20G 1.5″ needle. The peritoneal exudate cells were centrifuged for 10 min. at 400 × g, 4°C, and the pellet was resuspended in complete DMEM F12.

### Lysosomal membrane integrity

The integrity of the lysosomal membranes was assessed by the acridine orange (AO) relocation test as established previously 23. In brief, cells were first stained with AO and then underwent different treatments for different times, and then analysed by confocal microscopy. Cells with increased cytosolic/nuclear AO green fluorescence were identified as cells with lysosomal membrane permeabilization (LMP).

### Intracellular lipid

Cellular lipid levels were assayed by Oil red O (Sigma‐Aldrich) staining of cultured cells or sections from human carotid plaques. Briefly, cells or cryosections were fixed with 2% formalin, stained for 6 min. with 0.15% Oil red O in 76% methanol and 0.2 M NaOH, washed with water, counterstained with haematoxylin and visualized by light microscopy. Positive areas of oil Red O were analysed by Adobe Photoshop (v5.5) (Adobe Systems Inc., San Jose, California, USA).

### Human carotid atheroma

The Linköping Carotid Study is a prospective clinical‐pathology study in which atherosclerotic carotid arteries were collected from patients who underwent carotid endarterectomy at Linköping University Hospital. The ethics committee of Linköping University Hospital has approved this study as described previously 24, and informed consent was obtained from all participants.

Atherosclerotic carotid samples obtained from 15 patients (average‐age of 72) were included in this study. Ten patients with cerebrovascular symptoms were classified as symptomatic, and five patients without cerebrovascular symptoms were defined as asymptomatic. Several stroke risk factors were recorded, including hypertension (defined by hypertension history and diastolic blood pressure ≥110 mmHg, *n* = 10), smoking (smoking >5 years, *n* = 4) and diabetes mellitus (diabetes medication, *n* = 3). Carotid artery samples were collected immediately after endarterectomy. All operations were performed with minimal manipulation of the specimen without opening of the arterial lumen. The samples were divided into two parts: one part was directly frozen at −70°C and another part prepared for histopathology analysis after decalcification. Three to five transverse segments were taken from each specimen and fixed in 4% (w/v) formaldehyde and embedded in paraffin. These segments were sectioned with an interval of 2 μm.

### ApoE^−/−^CysC^+/+^ mice and apoE^−/−^CysC^−/−^ mice

Cryosections from the aortic sinus of atherosclerotic apoE‐deficient and CysC‐competent (apoE^−/−^CysC^+/+^) mice or apoE‐ and CysC‐deficient (apoE^−/−^CysC^−/−^) mice 19, which were fed a high fat diet, 21% cocoa fat, 0.15% cholesterol without sodium cholate (AnalyCen Nordic, Linköping, Sweden) for 25 weeks, were used for immunohistochemistry or TUNEL technique. The hearts and proximal aortas from the mice were fixed in Histochoice embedded in Tissue‐Tek (Sakura, Torrance, CA, USA) and stained as described below. All animal experiments were approved by the local animal care ethical committee.

### Apoptosis

In human carotid plaques or lesions from apoE^−/−^CysC^+/+^ and apoE^−/−^CysC^−/−^ mice, apoptotic cells were assayed by the TUNEL technique using TUNEL *in situ* cell death detection kit (Roche Molecular Biochemical, Mannheim, Germany). Cryosections were fixed in 2% PFA and permeabilized with 0.1% Triton x‐100 in 0.1% sodium citrate. The slides were incubated with TUNEL reaction mixture for 60 min. at 37°C in the dark followed by incubation with converter peroxidase (POD)/horse radish peroxidase (HRP) or with covert alkaline phosphatase (AP) for 30 min. at 37°C. Immunoreaction was visualized by 3,3′‐diaminobenzidine (DAB) for POD system or by fast red for AP system and counterstained with haematoxylin. Endogenous peroxidase was blocked with 3% H_2_O_2_ in methanol and unspecific background was blocked with 3% bovine serum albumin and 20% normal bovine serum in 0.1 M Tris‐HCl (pH 7.5). Control sections without TUNEL reaction mixture were run for each protocol, resulting in consistently negative results.

In cultured cells, apoptotic cells were detected by immunocytochemistry using an antibody recognizing activated caspase‐3 (BD PharMingen, Franklin Lakes, NJ, USA).

### Immunohistochemistry

Cryosections and formalin‐fixed paraffin embedded sections of human carotid plaques or cryosections from lesions of apoE^−/−^CysC^+/+^ and apoE^−/−^CysC^−/−^ mice were fixed in 2% PFA, permeabilized and incubated with rabbit primary antibodies: anti‐LC3β (4°C overnight), anti‐Atg5 (2 hrs at 22°C), anti‐CysC (4°C, overnight), anti‐p62/SQSTM1 (4°C, overnight), or anti‐ubiquitin (4°C, overnight). The sections were incubated with AP‐conjugated goat anti‐rabbit secondary antibody at 22°C for 1 hr and the immunoreaction was visualized by fast red. Other sections were incubated with HRP‐conjugated goat anti‐rabbit secondary antibody at 22°C for 1 hr, and the immunoreaction was visualized by DAB. Control sections without primary antibodies or with non‐immune IgG were run for each protocol, resulting in consistently negative results. The slides were counterstained with haematoxylin and visualized by light microscopy.

### Image analysis and classification of the plaques

All histological sections were examined under a light microscope, and the images were digitalized to a Macintosh computer with Image Grabber program (Toronto, ON, Canada). The microscope was set on the same parameters used to scan all samples. The plaques were classified into early and advanced plaques. Early lesions have an intact plaque with a fibrous cap >100 μm and have no lipid pool formation often with intact internal elastic membrane. Advanced lesions have intact or ruptured plaques with lipid pool formation, infiltration of leucocytes and a fibrous cap <100 μm.

The randomly digitalized images were analysed with Adobe Photoshop (v5.5) as described previously 24. Positively stained areas for each section were presented as immunopositive areas (%), which were calculated as the average immunostained area per pixel value divided by the total pixel value.

### Statistics

For statistical analysis, one‐way anova followed by a post hoc Newman–Keuls test were used for multiple comparisons. Correlation between CysC and LC3β or CysC and Atg5 in human carotid plaques was analysed by the Spearman correlation test and presented as the Spearman correlation coefficient (*r*). Results are given as mean ± S.E.M. *P* < 0.05 was considered statistically significant.

## Results

### Defective autophagy, reduced CysC and lipid oxidation in human atheroma

**Figure 1:**
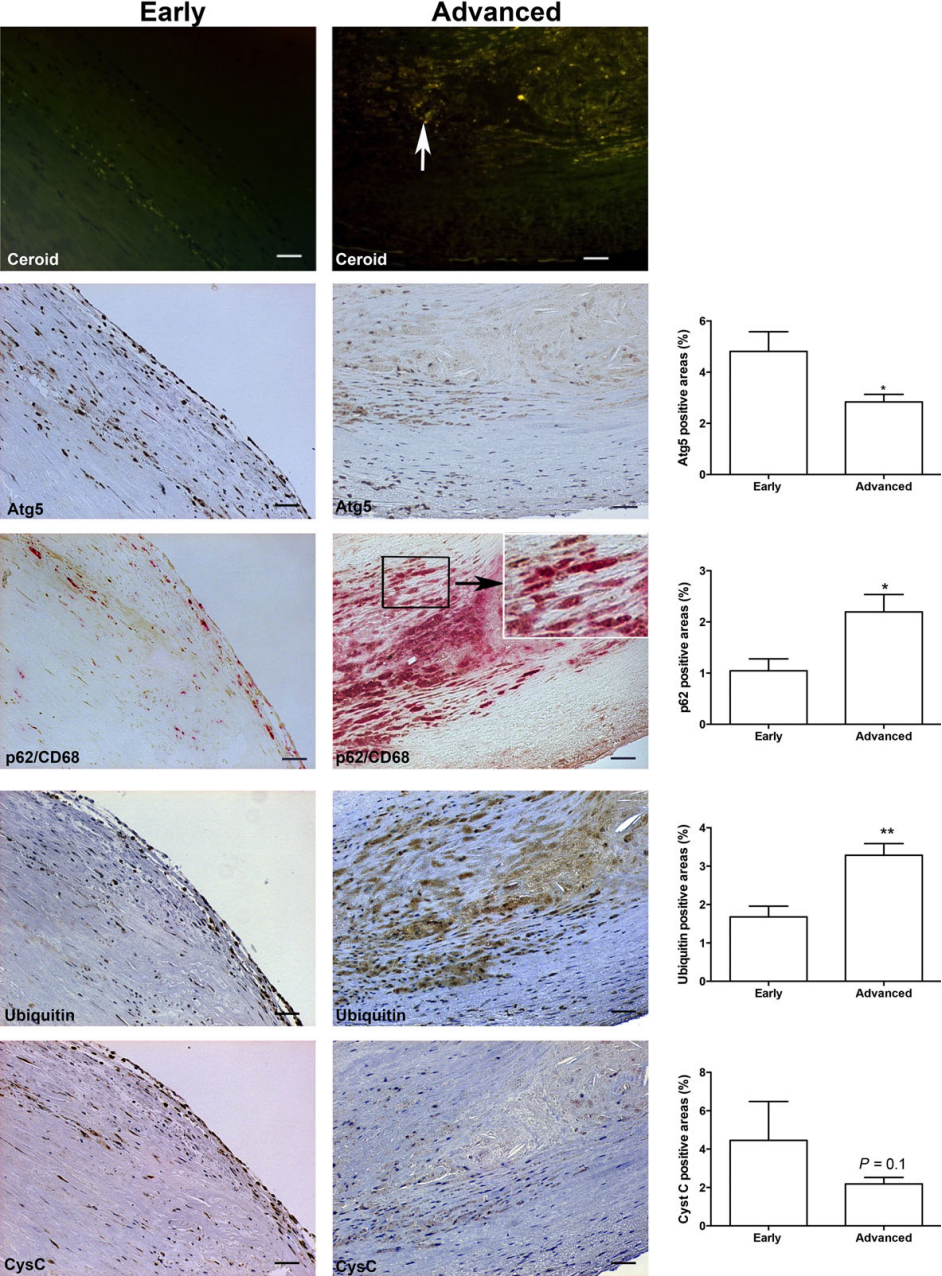
Decreased autophagy is associated with reduced CysC levels in progression of human atheroma. Representative photographs of autofluorescence of ceroid, Atg5 (brown), double immunohistochemistry of p62/SQSTM1 (brown) and CD68 (red), ubiquitin (brown) and CysC (brown) *in situ* in early lesions (left panels) and advanced human carotid lesions (middle panels); bars = 50 μm. *Note*: In early lesion areas, there are very low levels of ceroid, significantly higher levels of Atg5 and significantly lower levels of p62/SQSTM1 in CD68‐positive macrophages and low levels of ubiquitin, while advanced lesions show pronounced ceroid accumulation, low Atg5 and massive accumulation of CD68‐positive macrophages with significant increased levels of p62/SQSTM1 and ubiquitin and lower levels of CysC. Histograph show quantification of immunopositive areas of Atg5, p62/SQSTM1, ubiquitin and CysC. *P < 0.05, **P < 0.01.

Intracellular lipid accumulation is an important step in the progression of atherosclerotic lesions. Compared with early lesion of atherosclerosis lipid derived ceroid‐like material due to lipid oxidation is particularly abundant in areas rich in apoptotic macrophage foam cells in advanced lesions (Fig. 1, yellow auto‐fluorescence, white arrow).

To examine the relation between autophagy function and CysC expression in atheroma progression, Atg5, LC3β, SQSTM1, ubiquitin and CysC were examined by immunostaining in human carotid plaques from 15 patients. Atg5 expression was seen in all sections of both early and advanced lesions. In early lesions, Atg5 was mainly expressed in endothelial cells and intimal areas of the vessel wall (Fig. 1). In advanced lesions, the expression of Atg5 was significantly reduced, and in the areas close to the lipid rich areas, there were nearly no detectable levels of Atg5 (Fig. 1). As shown in Figure S1, Atg5 was mainly expressed in the fibrous cap and the expression of Atg5 appeared in cytoplasmic granules of macrophage foam cells (empty arrows) and in some endothelial cells (arrow with dotted line) and smooth muscle cells (arrow with solid line).

When autophagy flux is defective, p62/SQSTM1 and ubiquitin accumulate in lysosomes 25. Here, we found that the levels of p62/SQSTM1 and ubiquitin were significantly lower in early stages of human atherosclerotic plaques (Fig. 1). In contrast, massive and significant accumulation of p62/SQSTM1 and ubiquitin was observed in CD68‐positive macrophages in advanced lesions, especially near lipid‐rich areas (Fig. 1) suggesting deficiency of autophagy flux.

Expression of CysC, an inhibitor of lysosomal cathepsins, is decreased in human atheroma 18. To examine the relationship between CysC and autophagy in human atherosclerotic lesions, the expression of CysC was determined in human carotid samples. In early lesions of atherosclerosis, CysC expression was mainly seen in endothelial cells and in intimal areas (Fig. 1), which were positive for the autophagy marker Atg5, to some extent for p62/SQSTM1 and for ubiquitin (Fig. 1). In serial sections of advanced lesions, CysC expression was remarkably diminished together with massive accumulation of p62/SQSTM1 and ubiquitin (Fig. 1). Moreover, the expression of CysC was positively associated with the expression of both Atg5 (*r* = 0.76, *P* < 0.0001) and LC3β (*r* = 0.75, *P* < 0.0001).

### CysC deficiency is correlated with reduced autophagy and increased apoptotic cell death in atherosclerotic lesions of mice

**Figure 2:**
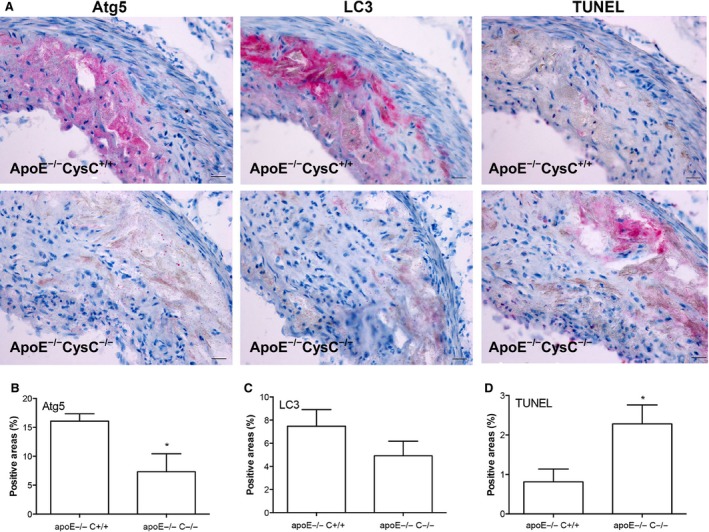
CysC deficiency in apoE^−/−^ mice results in reduced autophagy and increased apoptotic cell death. Lesions from apoE^−/−^CysC^+/+^ (*n* = 6) and apoE^−/−^CysC^−/−^ mice (*n* = 4) were immunostained with Atg5 or LC3β. Apoptosis was determined by the TUNEL technique, and images were analysed as described in the Methods section. The stained sections were counterstained with haematoxylin. (**A**) Representative photographs of Atg5, LC3β and TUNEL (stained in red) from apoE^−/−^CysC^+/+^ mice and apoE^−/−^CysC^−/−^, bars = 25 μm. (**B**–**D**) Quantification of Atg5, LC3β and TUNEL in the two groups of mice. *P < 0.05 *versus* apoE^−/−^ CysC^+/+^ mice.

Lack of CysC has been found previously to promote formation of large atheroma plaques with increased levels of lipid deposition in atherosclerotic apoE^−/−^ mice 19. Deficiency of CysC in apoE^−/−^ mice also resulted in an increased expression of lysosomal cathepsin B and L 26 that are involved in both autophagy and apoptosis 27, 28. To study whether the lack of CysC in mice had any effect on autophagy and cell death in atherosclerosis, we analysed Atg5 and LC3β for autophagy and TUNEL for apoptosis in lesions in apoE^−/−^CysC^−/−^ compared with apoE^−/−^CysC^+/+^ mice. Lesions in apoE^−/−^CysC^+/+^ mice showed strong immunostaining for Atg5 and LC3β and weak TUNEL reaction. Atg5 and LC3β were expressed mainly in the cytoplasm of cells present in the intima (Fig. 2A). In serial sections, only few red TUNEL‐positive cells were observed (Fig. 2A). On the contrary, lesions from apoE^−/−^CysC^−/−^ mice showed weak staining for Atg5 and LC3β and strong red TUNEL reaction (Fig. 2A). Quantification revealed significantly higher levels of Atg5 and lower levels of TUNEL in lesions from apoE^−/−^CysC^+/+^ mice compared with lesions from apoE^−/−^CysC^−/−^ mice (Fig. 2B–D).

### CysC prevents 2mix‐mediated LMP and apoptosis *via* autophagy pathways

**Figure 3:**
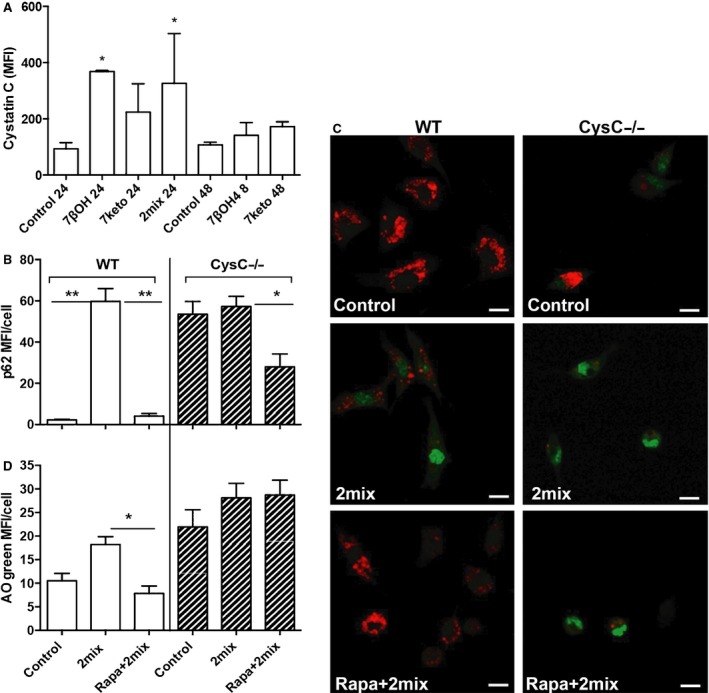
7‐oxysterols induce CysC and macrophages from CysC‐deficient mice (CysC^−/−^) show less autophagy activity and are more sensitive to 2mix‐mediated apoptosis by LMP. (**A**) THP‐1 cells were treated with 7‐oxysterol for 24 or 48 hrs, immunostained with CysC and analysed with flow cytometry (MFI: mean fluorescence intensity). *P < 0.05 *versus* control cells from the same time point (*n* = 2–6, mean ± S.D.). (**B**) Peritoneal macrophages from wild‐type (WT) or CysC^−/−^ mice were treated with 2mix for 12 hrs or pre‐treated with rapamycin for 1 hr and then exposed to the 2mix for 12 hrs, immunostained with anti‐p62/SQSTM1 antibody, photographed with confocal microscopy and quantified by image analysis. *Note*: in WT cells (white bars), p62/SQSTM1 was increased following 2mix exposure, which was reversed by rapamycin pre‐treatment (**P < 0.01). In CysC^−/−^ cells (hatched bars), basal levels of p62/SQSTM1 were already higher in untreated control cells as compared with the cells from WT mice. Furthermore, rapamycin pre‐treatment had less significant effect on 2mix‐induced p62/SQSTM1 in CysC^−/−^ cells (*P < 0.05). (**C** and **D**) Cells were first stained with lysosomal AO and then treated as indicated for 6 hrs and analysed by confocal microscopy (**C**, representative photographs of AO‐stains, bars = 10 μm), and cytosolic/nuclear AO green fluorescence intensity was analysed by Photoshop image analysis and was identified as cells with LMP (**D**, *P < 0.05). Note in **C**: Red fluorescence indicates AO located in lysosomes, whereas green florescence indicates relocation of AO from lysosomes to cytosol or nuclei.

Since CysC expression was positively associated with the autophagy markers Atg5 and LC3β in human carotid plaques, we examined whether exposure to 7‐oxysterols alters CysC expression in THP‐1 macrophages. We found that 7‐oxysterol treatment resulted in significant induction of CysC after 24 hrs, which was decreased to near the control levels after 48 hrs (Fig. 3A). At the same time point, 7‐oxysterol induced more than 2.07‐fold increase in cell death as compared with control cells as assayed by Annexin V/PI. We consider the induced CysC as a cellular self‐protective response, which, however, is often insufficient to protect cells from apoptotic cell death. In the same model, cell death–induced 7‐oxysterol was remarkably increased by autophagy inhibitor 3MA (194.7% that of 7‐oxysterol‐treated cells), while decreased by autophagy inducer rapamycin (62.8% that of 7‐oxysterol‐treated cells). To further understand the role of CysC in 2mix‐mediated apoptosis and autophagy, peritoneal macrophages from WT (CysC^+/+^) and CysC^−/−^ mice were exposed to the 2mix in the presence or absence of rapamycin. Immunointensity of p62/SQSTM1 was measured in 6–20 arbitrarily chosen 40× fields. Increased p62/SQSTM1 staining was found in macrophages of CysC^−/−^ as compared with control cells of WT mice (Fig. 3B). Following exposure to 2mix, p62/SQSTM1 was significantly increased in WT cells, whereas in CysC^−/−^ cells, there are already high levels of p62/SQSTM1 in untreated CysC^−/−^ cells (Fig. 3B). Rapamycin significantly diminished 2mix‐mediated accumulation of p62/SQSTM1 in WT cells, while it had less effect on CysC^−/−^ cells (Fig. 3B), indicating that CysC deficiency is associated with dysfunctional autophagy.

We next studied LMP and apoptosis in macrophages isolated from WT and CysC^−/−^‐deficient mice by AO relocation test and assay of active caspase 3. AO green fluorescence was measured in 14–20 arbitrarily chosen 40× fields, and cells with increased cytosolic/nuclear AO green fluorescence were identified as cells with LMP. Untreated WT macrophages showed typical lysosomal AO red fluorescence and low cytosolic/nuclear AO green fluorescence, whereas untreated CysC^−/−^ macrophages showed lower levels of lysosomal AO red fluorescence and more than a twofold increase of cytosolic/nuclear AO green fluorescence (Fig. 3C and D), indicating vulnerability of lysosomal membranes in CysC^−/−^ cells. Importantly, rapamycin had a significant protective effect on LMP induced by 2mix in WT cells, whereas no such effect of rapamycin was seen in CysC^−/−^ macrophages (Fig. 3C and D), indicating that the cytoprotective effect of autophagy is dependent on CysC.

After 24 hrs, there was no obvious difference in caspase 3 activity between untreated CysC^−/−^ cells (MFI = 8.7 ± 3.8) and untreated WT cells (MFI = 9.6 ± 4.5). Following 2mix exposure, a more than fourfold increase in active caspase 3 was detected in WT cells (MFI = 38.9 ± 14.9), while a more than ninefold increase in active caspase 3 was found in 2mix‐treated CysC^−/−^ cells (76.2 ± 4.4), suggesting that cells from CysC^−/−^ mice are more sensitive to 2mix mediated apoptosis or in another word CysC protects against 2mix‐mediated apoptosis.

**Figure 4:**
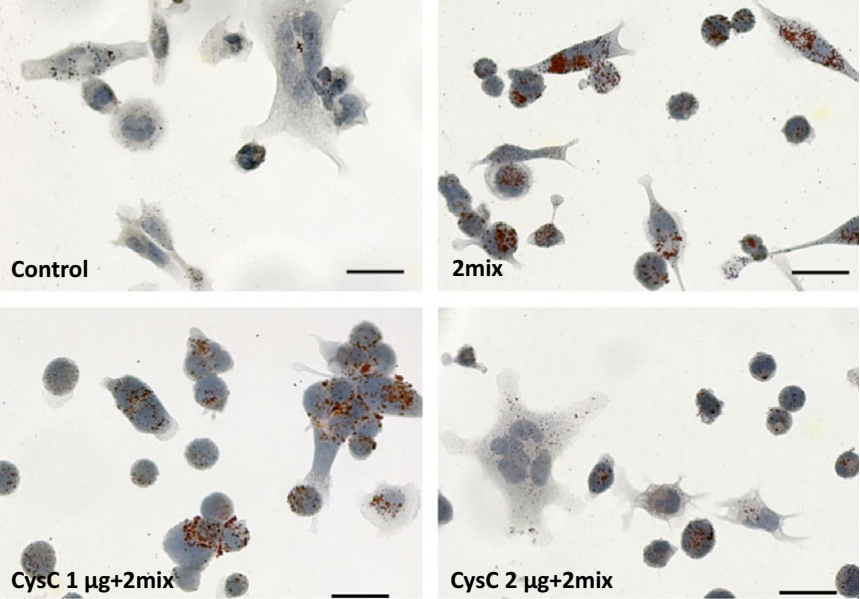
2mix‐induced lipid accumulation is minimized by 2 μg human recombinant cystatin C. THP‐1 differentiated macrophages were treated with or without 2mix for 24 hrs in the presence or absence of CysC. Cells were stained with Oil red O and counterstained with haematoxylin and analysed by light microscopy; bars = 20 μm. Percentage of oil Red O positive areas: control 0.55 ± 0.16, 2mix 3.47 ± 0.55, CysC 1 μg + 2mix 3.20 ± 0.61 and CysC 2 μg + 2mix 0.80 ± 0.07.

We have previously shown that atheroma‐related 7‐oxysterols cause cellular lipid accumulation in monocytes 20. To examine whether CysC can rescue 2mix‐induced lipid accumulation and cell damage, THP‐1 differentiated macrophages were treated with human recombinant CysC together with 2mix for 24 hrs. Cellular lipid was assayed by oil red O and nuclear/cell morphology by haematoxylin staining. As shown in Figure 4, lipid accumulation induced by oxysterols was minimized in the presence of 2 μg/ml CysC. The quantified positive areas of oil red O is presented in Figure 4. Moreover, numbers of shrunken cells with condensed nuclear staining were reduced by 2 μg/ml of CysC.

## Discussion

Oxysterol accumulation and related cell death are of importance in necrotic core formation and progression of atherosclerotic plaques. Our results demonstrate that dysfunctional autophagy is a characteristic of advanced human atherosclerotic lesions, which is associated with lipid oxidation and inversely associated with CysC expression. *In vitro*, CysC plays a regulatory role in autophagy that protects against apoptosis in macrophages induced by 7‐oxysterols *via* preventing LMP and lipid accumulation.

Both apoptosis and autophagic cell death play pivotal roles in plaque rupture and thrombosis of atherosclerotic lesions. However, the relationship and molecular interplay between CysC, autophagy and apoptosis have not yet been investigated in atherosclerotic lesions. We report here that the inverse association between autophagy activity and human atheroma progression suggest that the autophagy activity in early human atheroma lesions may be a transient self‐defence, which then declines following prolonged lipid oxidation and oxidative stress. Our data for the first time demonstrated that autophagic flux through lysosomes decreases as plaques progress in advanced human atheroma lesions. A similar reduction of autophagy activity has previously been only reported in the failing human heart 29 and a mouse model of coronary artery occlusion 30. Our findings imply that autophagy activity may play a crucial role in maintaining atheroma plaque stability *via* regulation of lipid accumulation and necrotic core formation. The mechanism may involve a decrease in cholesterol efflux by cells due to the lack of Atg5, which previously has been shown to augment lipoprotein cholesterol ester retention 31.

CysC is an endogenous cysteine protease inhibitor, which has a broad spectrum of biological roles, including cell proliferation and modulation of inflammatory responses. In cardiovascular disease, expression of CysC is decreased in human atherosclerotic lesions 18 and lack of CysC enhances atherosclerosis 19. One of the anti‐atherosclerotic functions of CysC is the inhibition of lysosomal cathepsin that is significantly associated with apoptosis and plaque destabilization in human atherosclerosis 24. Our findings show a significant positive association between autophagy and CysC expression in human carotid plaques, but an inverse correlation between autophagy and apoptosis in lesions in CysC‐deficient apoE^−/−^ mice, proposing a new function for CysC as an autophagy regulator in atherosclerosis. Furthermore, the cytoprotective effect of CysC, as shown in 7‐oxysterol‐treated cells in the presence or absence of rapamycin, is likely to be mediated through the induction of functional autophagy. More interestingly, the reduction of cellular lipid accumulation mediated by CysC in our study suggests an undiscovered function of CysC. However, further studies are necessary for understanding the mechanisms.

In conclusion, dysfunctional autophagy is characteristic of advanced human atherosclerotic lesions. Moreover, autophagy is positively associated with CysC expression and inversely associated with progression of the lesions. Our findings raise the possibility that CysC expression and effective autophagy activity may serve as a safeguard in combating oxidized lipid‐mediated cytotoxicity to limit necrotic core formation in atheroma progression. The findings call for further studies on autophagy and CysC in ApoE^−/−^CysC^−/−^ mice and large clinical settings.

## Funding

The study was supported by grants from the Swedish Heart Lung Foundation, the Torsten and Ragnar Söderbergs Foundation, the Stroke Foundation, the Olle Engkvist Foundation, the Swedish Gamla Tjänarinnor Foundation, the Linköping University and Linköping University Hospital Research Foundation, the Magnus Bergvall Foundation, Syskonen Svensson Foundation, the Crafoord Foundation, the Royal Physiographic Society, the Lars Hierta Foundation, the Lundström Foundation, the Malmö University Hospital Foundation, the Swedish Society of Medicine and the USA National Institutes of Health.

## Conflict of interest

The authors confirm that there are no conflicts of interest.

## Supporting information


**Figure S1** Increased lipid accumulation and apoptosis in advanced human atheroma.
**Figure S2** Expression pattern of Atg5 in an advanced human carotid plaque.
**Figure S3** Decreased LC3β in advanced human atheroma.
**Figure S4** CysC expression positively correlates with the expression of Atg5 and LC3β assayed by Spearman correlation coefficient test.
