# Supplementary material for: Autophagy dysfunction and regulatory cystatin C in macrophage death of atherosclerosis
Source: J Cell Mol Med. 2016 Apr 14;20(9):1664–72. doi: 10.1111/jcmm.12859 (PMC4988293; doi:10.1111/jcmm.12859)
Supplement: Supplementary file 1 — Figure S1 Increased lipid accumulation and apoptosis in advanced human atheroma. Figure S2 Expression pattern of Atg5 in an advanced human carotid plaque. Figure S3 Decreased LC3β in advanced human atheroma. Figure S4 CysC expression positively correlates with the expression of Atg5 and LC3β assayed by Spearman correlation coefficient test. [file JCMM-20-1664-s001.ppt]

## Slide 1
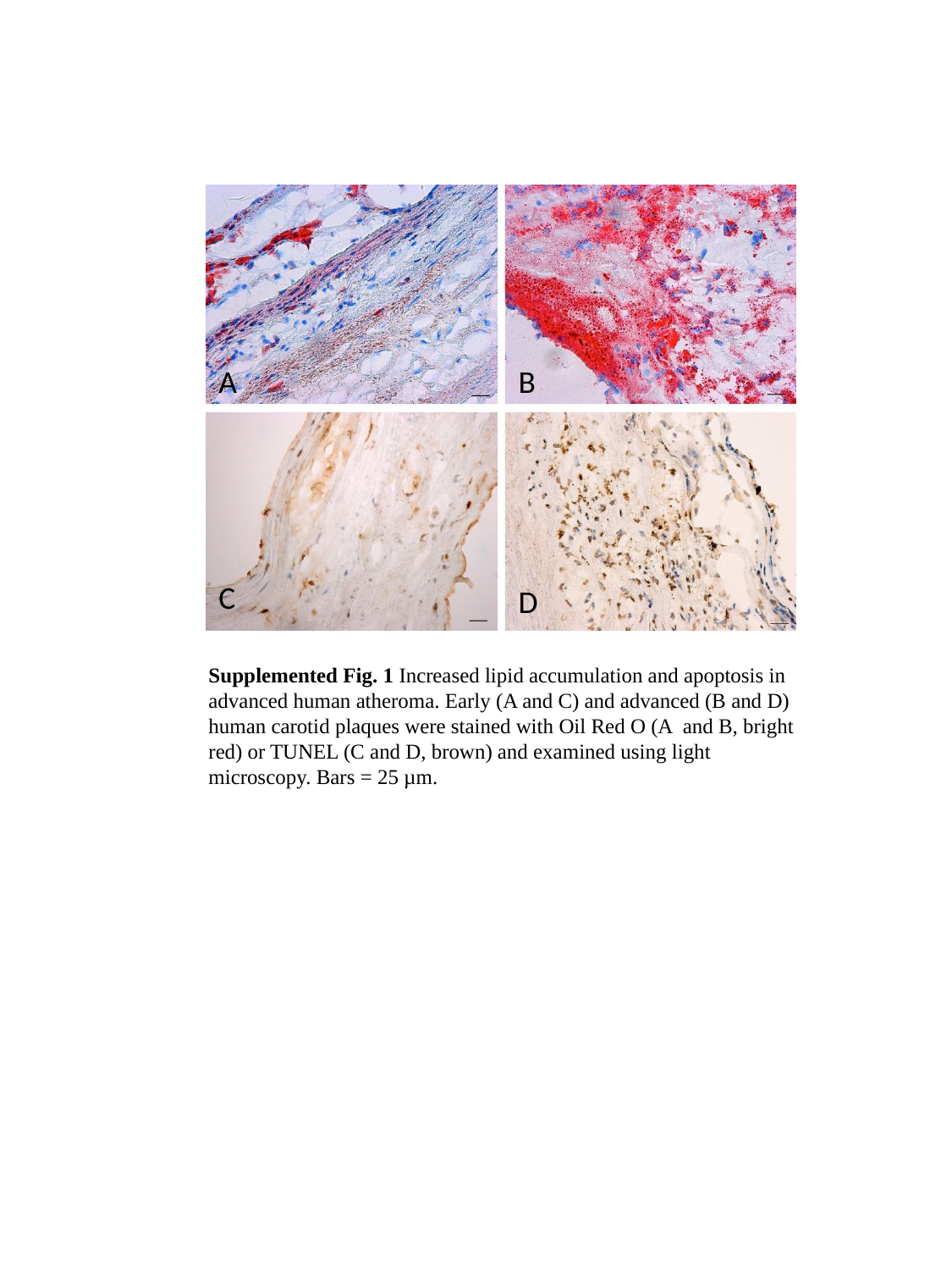

A
B
C
D
Supplemented Fig. 1 Increased lipid accumulation and apoptosis in advanced human atheroma. Early (A and C) and advanced (B and D) human carotid plaques were stained with Oil Red O (A and B, bright red) or TUNEL (C and D, brown) and examined using light microscopy. Bars = 25 µm.

## Slide 2
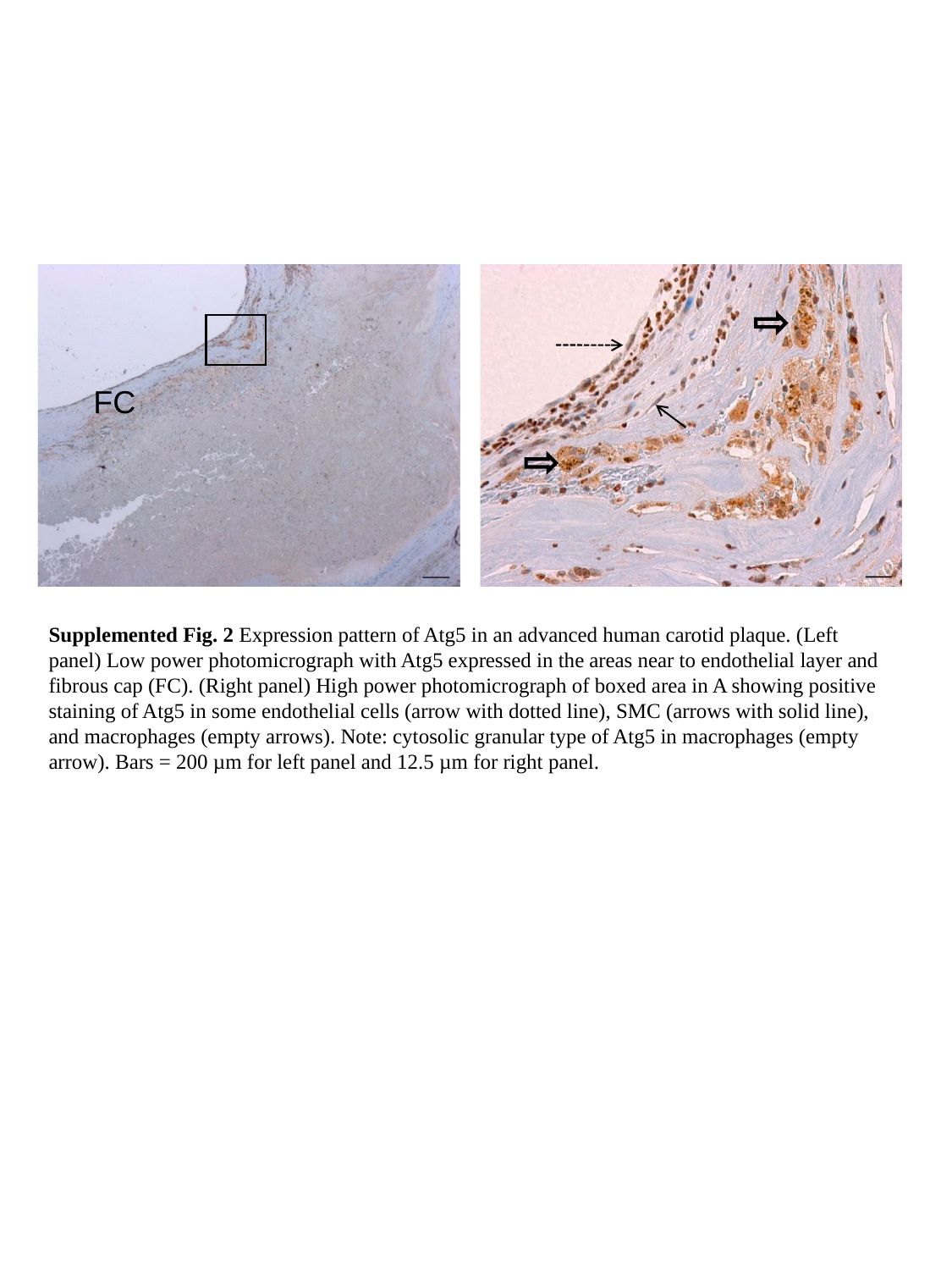

FC
Supplemented Fig. 2 Expression pattern of Atg5 in an advanced human carotid plaque. (Left panel) Low power photomicrograph with Atg5 expressed in the areas near to endothelial layer and fibrous cap (FC). (Right panel) High power photomicrograph of boxed area in A showing positive staining of Atg5 in some endothelial cells (arrow with dotted line), SMC (arrows with solid line), and macrophages (empty arrows). Note: cytosolic granular type of Atg5 in macrophages (empty arrow). Bars = 200 µm for left panel and 12.5 µm for right panel.

## Slide 3
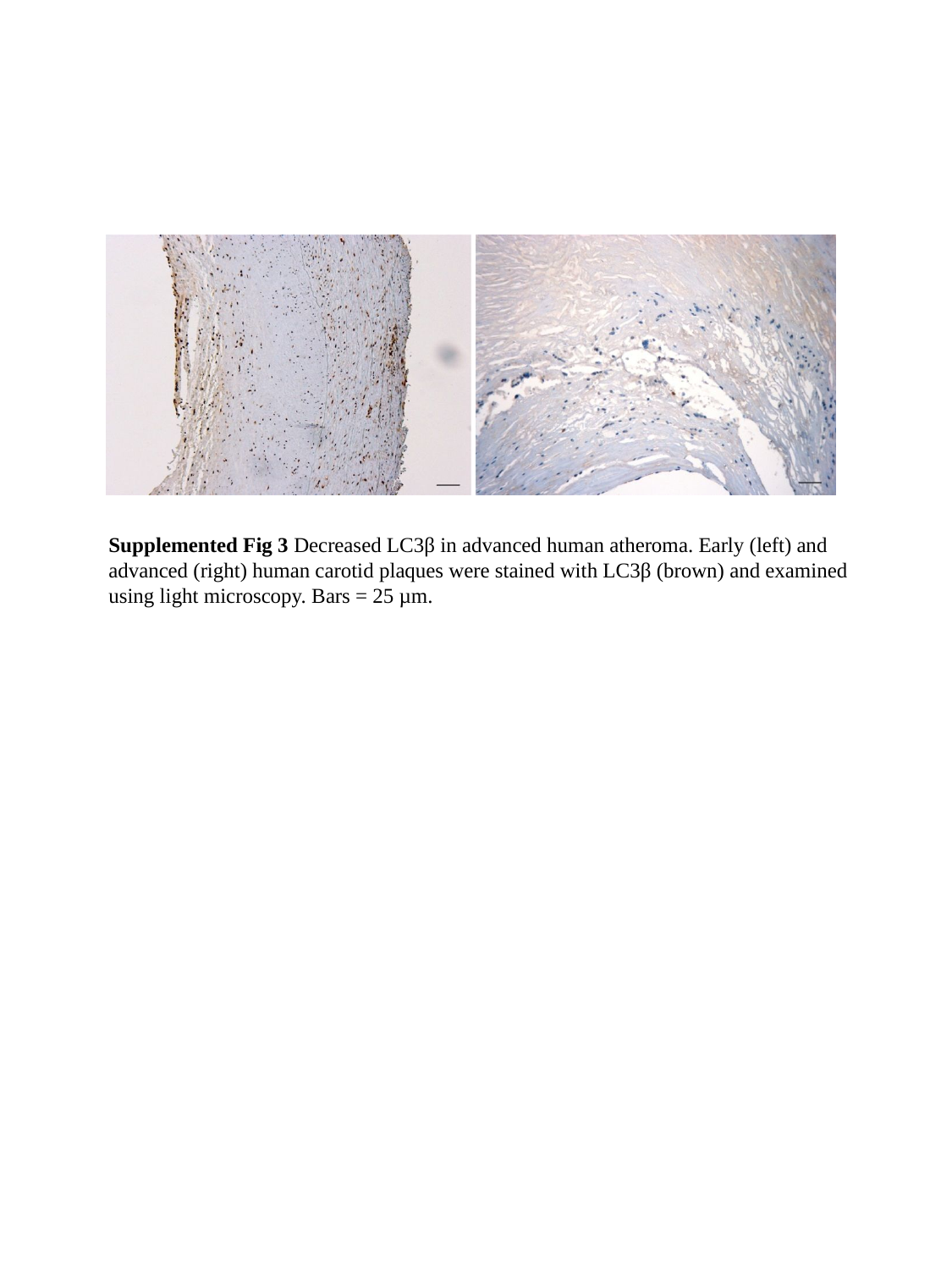

Supplemented Fig 3 Decreased LC3β in advanced human atheroma. Early (left) and advanced (right) human carotid plaques were stained with LC3β (brown) and examined using light microscopy. Bars = 25 µm.

## Slide 4
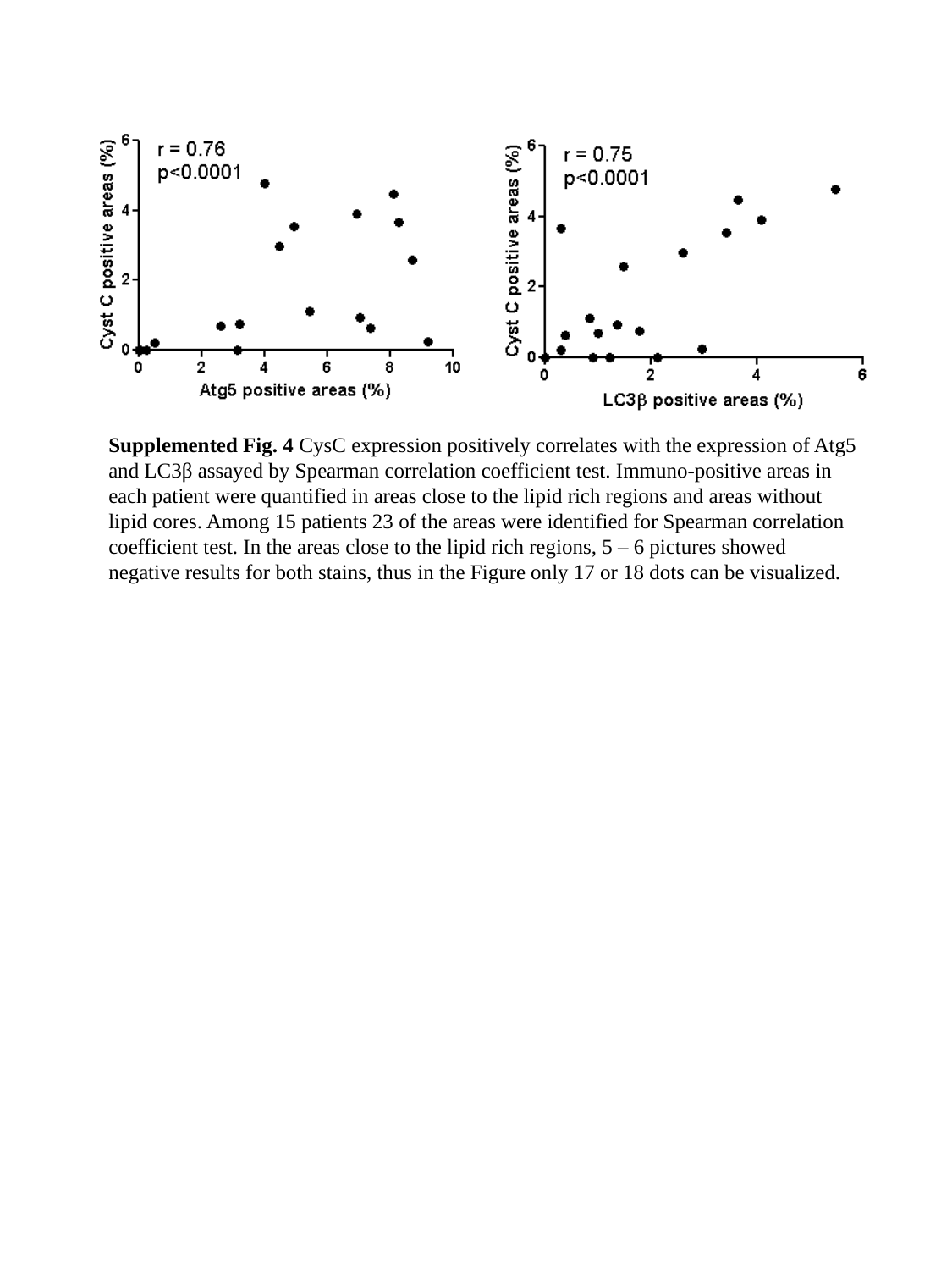

Supplemented Fig. 4 CysC expression positively correlates with the expression of Atg5 and LC3β assayed by Spearman correlation coefficient test. Immuno-positive areas in each patient were quantified in areas close to the lipid rich regions and areas without lipid cores. Among 15 patients 23 of the areas were identified for Spearman correlation coefficient test. In the areas close to the lipid rich regions, 5 – 6 pictures showed negative results for both stains, thus in the Figure only 17 or 18 dots can be visualized.
